# Optimizing biomedical discoveries as an engine of culture change in an academic medical center

**DOI:** 10.1017/cts.2021.888

**Published:** 2022-01-02

**Authors:** Anne K. DeChant, Stephen Fening, Michael Haag, William Harte, Mark R. Chance

**Affiliations:** 1 Office of Translation and Innovation, Clinical and Translational Science Collaborative, School of Medicine, Case Western Reserve University, Cleveland, OH, USA; 2 Department of Biomedical Engineering, Case Western Reserve University, Cleveland, OH, USA; 3 Office of Research and Technology Management, Case Western Reserve University, Cleveland, OH, USA; 4 Department of Nutrition, Center for Proteomics and Bioinformatics, School of Medicine, Case Western Reserve University, Cleveland, OH, USA

**Keywords:** Innovation, translational research programs, workforce development, entrepreneurship, training, academic ecosystem, biomedical technologies

## Abstract

Academic discovery in biomedicine is a growing enterprise with tens of billions of dollars in research funding available to universities and hospitals. Protecting and optimizing the resultant intellectual property is required in order for the discoveries to have an impact on society. To achieve that, institutions must create a multidisciplinary, collaborative system of review and support, and utilize connections to industry partners. In this study, we outline the efforts of Case Western Reserve University, coordinated through its Clinical and Translational Science Collaborative (CTSC), to promote entrepreneurial culture, and achieve goals of product development and startup formation for biomedical and population health discoveries arising from the academic ecosystem in Cleveland. The CTSC Office of Translation and Innovation, with the university’s Technology Transfer Office (TTO), helps identify and derisk promising IP while building interdisciplinary project teams to optimize the assets through key preclinical derisking steps. The benefits of coordinating funding across multiple programs, assuring dedicated project management to oversee optimizing the IP, and ensuring training to help improve proposals and encourage an entrepreneurial culture, are discussed in the context of a case study of therapeutic assets, the Council to Advance Human Health. This case study highlights best practices in academic innovation.

## Introduction

Academic medical centers and their lead universities in the United States are recipients of tens of billions of dollars in Federal and Foundation funds [1] to study, understand, and alleviate human suffering to ultimately prevent, treat, and cure disease. In order for these discoveries to have the opportunity to reach the market and have patient impact, patent filings and studies into the safety and efficacy of the discovery need to be completed to industry standards. Since the advent of the Patent and Trademark Law Amendments Act (e.g. Bayh-Dole act) in 1980 [2], nonprofits and small businesses have had the right to retain ownership in inventions developed under Federal contracts, leading to an increase in academic patent filings in many fields at American research universities [3,4].

In the regulated world of biomedical products—drugs, diagnostics, devices, and digital health—the costs to develop products and assure their safety and efficacy can take millions to hundreds of millions of dollars and many years [5,6]. These levels of funds are beyond the capacity of individual nonprofit institutions. Thus, University and Hospital approaches to developing products require active and ongoing connections to investors and industry to engineer a successful hand-off of the technology assets to provide ultimately a positive impact on society as well as enhance the institution’s reputation for innovation [7,8,9].

**Fig. 1.**
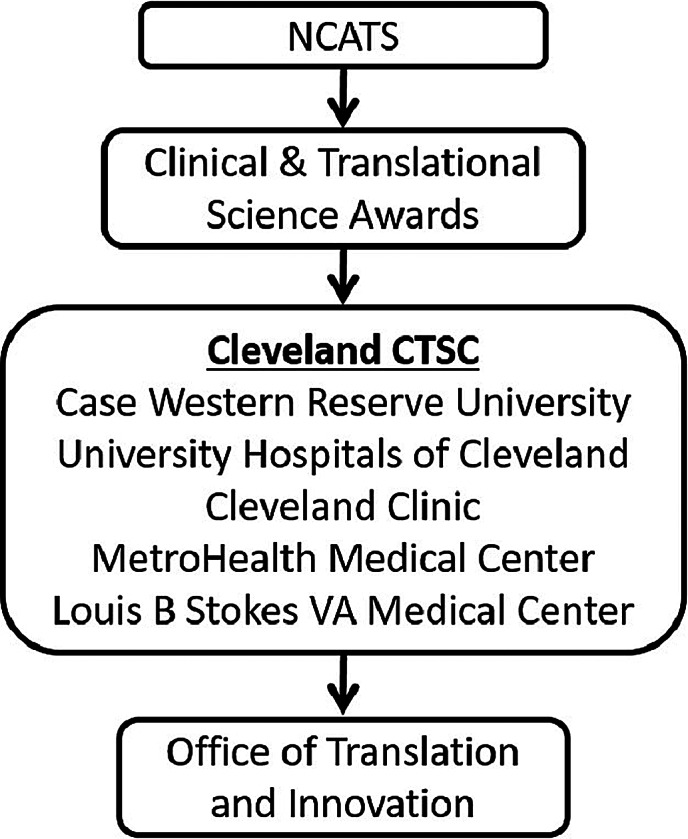
The Cleveland CTSC system. The National Center for Advancing Translational Sciences (NCATS) supports Clinical & Translational Science Awards (CTSA) at more than 50 institutions across North America. The Cleveland Clinical & Translational Science Collaborative (CTSC) consists of Case Western Reserve University along with four regional hospital systems. Within the Cleveland CTSC, the Office of Translation and Innovation helps support the ecosystem of entrepreneurship and innovation in northeast Ohio.

In this study, we will outline the efforts of Case Western Reserve University, coordinated through its Center for Translational Science Collaborative (CTSC) Office of Translation and Innovation, to further the goals of product development and startup formation for drugs, devices, diagnostics, and digital health technologies derived from the academic and hospital laboratories. The CTSC, funded by the National Center for Advancing Translational Sciences (NCATS), has as its mission to “catalyze the generation of innovative methods and technologies that will enhance the development, testing and implementation of diagnostics and therapeutics across a wide range of human diseases and conditions.” In Cleveland, Case Western Reserve University serves as the CTSC hub organization, connecting multiple affiliate hospitals (Fig. 1) to form a network of engineers, physicians, and scientists thousands strong with hundreds of millions of dollars per year in federal, Ohio, regional, and philanthropic support for research which helps maintain a vibrant discovery pipeline. Across the ecosystem, pilot funding and workforce development activities (including those inside and outside the CTSC) are coordinated by an Office of Translation and Innovation (OTI), which includes stakeholders from across campus, hospital affiliates, and alumni.

OTI, by including a wide range of stakeholders, can help coordinate multiple sources of pilot funding, project management, and entrepreneur support. This can result in multiple rounds of milestone driven and coordinated funding and advice at the technology optimization stage. OTI also helps coordinate entrepreneurship training and networking for translational teams. The major conclusion from our main case study of therapeutics, the Council to Advance Human Health, is that investigators are quite receptive to, and can be successful in participating in translating their discoveries. However, scientists require strong support in terms of respected outside advice and milestone-driven pilot funding, attentive project management, continued education around market need, and encouragement to pivot when indicated. Given these kinds of stable, consistent support, an entrepreneurial culture of academic discovery can be developed within 10 years, given sustained effort and resources that include access to translational funding, training opportunities for program teams, and broad collaboration and coordination between various translational research stakeholders, university leadership, and regional partners. Immediate benefits to institutions undertaking these efforts can include faculty and student attraction and retention, interdisciplinary team building, increased federal and foundation grants, increased intellectual property disclosures and patents, participating unit and institutional visibility, patient impact, new businesses launched, and over time, licensing returns to help sustain the effort.

## Methods and Results

### Case Coulter Translational Research Partnership

CWRU’s research portfolio is relatively large befitting the University’s status as an R1 institution with a medical school in the top 20 funding nationally, including a very strong biomedical engineering department. To optimize these discoveries, the Case Coulter Translational Research Partnership was established in 2006 as the first organized and sustained biomedical accelerator program at the university (Table 1). In Coulter, a biomedical engineer is teamed up with a clinician, with a goal of optimizing the technology towards an unmet medical need. A critical question about any potential Coulter program when considered for funding always includes, can this technology be sufficiently developed to be licensed to industry or become a viable startup within 3 years. Coulter has achieved exits for its technologies consistent with this time scale for a significant fraction of the supported projects. Along the way, the technology optimization teams have achieved additional funding for the technologies in the form of federal and foundation grants, and when the technologies have been turned in to companies, those companies have attracted substantial dilutive capital as well. Over the life of the Case-Coulter program, 69 projects have received full program support (>$25,000) and many others have received pilot awards. A total of 33 technologies have been licensed, with a total of 38 licenses. This is an overall licensing rate of 48%.

**Table 1.** Translational funding programs

	Case-Coulter Translational Research Partnership	Council to Advance Human Health	Clinical & Translational Science Collaborative	NIH Center for Accelerated Innovations	Ohio Third Frontier	Taipei Medical University
Focus	Biomedical Engineer and clinical partner	Therapeutics		National Heart Lung & Blood Institute	Software/Information Technology, Biomedical/Life Sciences (other)	Neurology, oncology, AI, aging
Funding	$25,000–$250,000	$50,000–$100,000	$50,000	$75,000–$125,000	$50,000	$50,000
Frequency	Annual	Annual	Annual	3× per year	3–4× per year	Annual
Awards	6	3	5	4	3	5
Year started	2006	2012	2002	2013	2016	2016

The total amount invested from the Case-Coulter program over the history of the program is $11.5M. As of the end of 2020, these technologies have raised more than $290M of funding after inclusion in the program (follow-on funding). Approximately $160M has come in the form of dilutive investment to the companies and $120M has come in the form of grants to either the university or companies in support of these technologies. This is a follow-on-funding ratio of 25:1. Twenty-eight startup companies have been formed around these technologies, most of them in Ohio. Forty technologies are available for human use. These data provide the benchmarks for developing our other programs and were especially helpful in establishing the Council to Advance Human Health.

### Council to Advance Human Health: A Case Study of Therapeutics Acceleration

The School of Medicine developed the Council to Advance Human Health (CAHH) beginning in 2012 as a focused therapeutics accelerator, with similar principles of milestone driven funding and industry-based advice and alignment in product development activities as in the Coulter program. Although the goals of licensing and/or startup for therapeutics within 3 years were considered challenging, we have found that once pre-clinical proof of concept was established, a 3-year time line for exit or license was reasonable for many assets.

CAHH started with the hiring of translational officers tasked to be technology scouts and to evaluate and project manage funded projects. External contractors, one with 25+ years’ experience in the pharmaceutical industry and the other a serial start-up entrepreneur, were brought on board part time to provide advice on the value of the IP portfolio and to build relationships with faculty. These translation officers were connected to the Technology Transfer Office Intellectual Property portfolio and collaboratively with licensing managers and investigators created action plans for the best and brightest technologies.

Funding issues were addressed by creating a philanthropic accelerator fund. Alumni were asked to contribute to a general accelerator fund instead of specific endowments or projects. Funds were provided as milestone-driven awards for non-discovery activities, with the expectation that the money would be spent quickly, often using outside contract organizations to perform the necessary developmental experiments. The Dean of the School Medicine at the time contributed funds from the school’s catalytic fund to help jumpstart the funding. In addition to the monetary contributions, specific alumni were convened as a council of Discovery Experts, who were presented with the best therapeutics technologies and plans for their development in sessions with the investigators. The sessions provided feedback and advice, and helped connect the teams to potential other partners and investors. The teams also received CAHH awards and immediately pursued product development activities, either by testing pre-clinical models, enhancing IP, or developing regulatory strategies and business plans.

Many CAHH programs went on to receive funding from other TRPs, like NCAI—the NHLBI pilot translational accelerator, Coulter, or the Ohio Third Frontier—the state funding organization focused on economic development in Ohio. Each time a program received an award, progress was tracked and managed by the Office of Translation and Innovation. This permitted a series of independent but related questions to be answered in the product development timeline for the therapeutic, further enhancing valuation and reducing risk. These changes sparked real excitement in innovation, and in the following years, projects became more focused and mature. Discussions at council meetings were constructive, and helped create sharper, more thoughtful programs. The Technology Transfer Office began to see more interest from companies looking to license technologies, and investigators were bringing more developed ideas to their office.

Over the 9 years in the program to date, the results are remarkable (Table 2). The Accelerator Fund invested $1.5M in 20 programs resulting in eight new licensing deals, two clinical trials, and over $150 million in outside investment in CWRU-based technologies to date. In addition, over $55 million in translational grants to investigator laboratories were received. This total return dwarfs even our impressive Coulter numbers, which were 25:1 for dilutive and non-dilutive return, here they are exceeding 130:1 so far. However, the total potential earnouts exceed $1 billion at present, if milestones relating to clinical development are met. Thus the impact of the project, even though it is already higher than expected, may be mostly ahead. It should be emphasized that the total costs of the program, e.g. translational officers and other consultants, plus faculty time on projects, far exceed the specific project costs of the TRP awards. The returns above can be achieved only when the project investments are made in the context of an ecosystem with significant maturity and devoted resources. Notably, many of the CAHH programs received support from multiple sources within the innovation ecosystem.

**Table 2.** Council to Advance Human Health (CAHH) funded projects and outcomes including company formed, clinical trials, and Pharma partnerships

Date	CAHH programs	Company formed	IP licensed	Clinical trials	Follow on Pharma partner
2012	Alzheimer’s disease therapeutic	ReXceptor*	2013	Ph1 2013	
2012	Metastasis detection and prognosis	NeoIndicate	2020		
2012	Small molecules for triple (-) breast cancer				
2012	Photodynamic therapy for skin disease	Fluence Therapeutics*			
2013	Protein tyrosine phosphatase (PTP)-sigma peptide for tissue regeneration	NervGen	2018	Ph1 2021	
2014	Remyelination therapeutics for Multiple Sclerosis	Convelo Therapeutics	2017		Genentech (2019)
2014	Anti-virulence agent against methicillin-resistant *Staphylococcus aureus* (MRSA)	Q2 Pharma	2017		
2014	15-Hydroxyprostaglandin Dehydrogenase (PGDH) inhibitor for tissue regeneration	Rodeo Therapeutics	2017		Amgen (2021)
2015	Small molecule for inflammation				
2015	Ribonucleotide Reductase modulators for pancreatic cancer treatment				
2015	Apoptosis regulator for cancer treatment				
2016	Virus nanoparticles for cancer immunotherapy	Mosaic Immunoengineering	2020		
2016	Peptide and small molecule inhibitors of Huntingdon’s disease				
2017	Synthetic platelets for hemorrhage	Haima Therapeutics	2020		
2017	Phosphatases in tumorigenesis				
2018	tRNA suppression to treat Dravet and other epilepsies	Tevard Biosciences	2019		Zogenix (2020)
2018	Gene therapy treatment for haplo-insufficiency diseases				
2019	Osteosarcoma immunotherapy				
2019	Myeloid-related protein 14 (MRP14) antibody for Lupus				
2020	B-cell activating factor chimeric antigen receptor Natural Killer (BAFF CAR-NK) cells to treat B-cell cancers				

* Licensed prior to CAHH award support.

In terms of specific Pharma partnerships, Convelo entered a Collaboration and Option to Acquire Agreement with Genentech in 2019 on undisclosed confidential terms and Rodeo Therapeutics was acquired by Amgen in 2021 for $55 million, with potential additional milestone payments of $666 million possible (Table 2). This is remarkable considering that both programs were evaluated and funded by CAHH in 2014. By 2017 both had been launched into university startup companies and quickly achieved Series A rounds of over $5 million. A third CWRU startup company, Tevard Biosciences, has signed a major licensing deal with Zogenix, with significant potential for follow-up on funding and return (Table 2). Each of these deals assures that if the discoveries are found to be safe and effective, resources necessary to bring them to patients are likely to be available. Without such resources, our discoveries will never get to market or have a patient impact. Further, all the CAHH awardees have advanced their research programs in the lab with follow-on grants and foundation awards. The CAHH program is currently focused on increasing understanding of regulatory pathways and obstacles, in order to further enhance the speed of translation.

### Office of Translation and Innovation

As the university’s ecosystem matured, gained technology optimization funding programs (Table 1) and later added entrepreneur training programs, we had needed to coordinate and exchange information about entrepreneurs and projects to help provide the best advice for development of technologies and to give those discoveries the best opportunity to achieve external funding and potentially reach the market. Further, we needed to track outcomes across multiple programs to understand the inputs and outputs for the innovation ecosystem. The Office of Translation and Innovation (OTI) was launched in 2012 with a vision to accelerate the movement of technologies from lab toward societal impact and a mission to directly engage faculty and programs to coordinate activities and improve efficiencies of developing products. With both CTSC and institutional support, the OTI became the coordinating office for translational research program funding and training. We established it with a Director (Chance) and Executive Director (DeChant), a support staff involved in reviewing, awarding, project managing, and tracking CTSC and other related pilot grant funding programs while using an amalgamation of funding including CTSC, University, and other available resources. DeChant and Chance led monthly meetings, inviting interested stakeholders from the institution and beyond. These stakeholders included representatives from the Technology Transfer Office, the translation officers and scouts, other biomedical accelerator funding programs associated with the University and its affiliates, CTSC leadership, development office personnel involved in translational fundraising, and CWRU faculty and staff leading programs that develop technology or support entrepreneurs, including those at the Cleveland Clinic and the VA (Table 1). The monthly meetings became a venue for identifying promising inventors and technologies engaged with multiple stakeholders, as well as a venue for establishing additional collaborations across the state (e.g. NCAI) and across the world (Taipei Medical University). Much of the meeting time became devoted to understanding the needs and timing of support required to speed the development of these high value products, either towards license or startup.

**Fig. 2.**
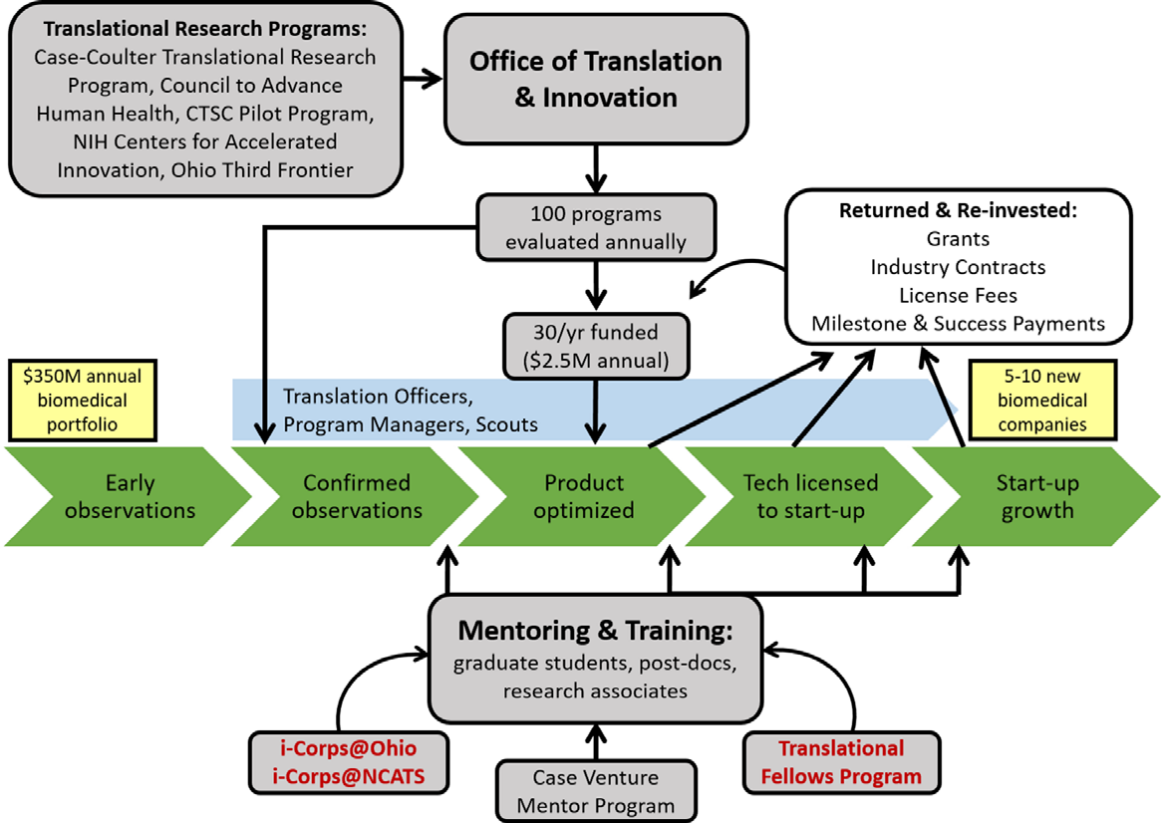
Translational research ecosystem in Cleveland. The figure shows the five major stages of innovation and product development from discovery through technology optimization, startup, clinical approvals through societal impact in the context of growing and developing new companies (green). Gray boxes indicate major inputs represented as translational research programs and entrepreneurial services, including the Clinical and Translational Science Collaborative (CTSC). Outputs to Case Western Reserve University (CWRU) occur as new grants during the optimization process and new funds back to CWRU in terms of license fees and milestone payments as startups form and progress. A gray box also denotes major outputs, represented as the drugs, devices, and medical and health interventions that impact patients. Red lettering indicates new programs (2020), including two i-Corps programs – innovation programs using experiential education in entrepreneurship.

In Fig. 2, we show the five stages of the CWRU biomedical academic pipeline (green) and OTI’s location within the ecosystem. The Translation Research Programs (TRPs) of Table 1 are seen in the top left, they provide funding to projects with assistance in advice, tracking, and coordination though OTI. The TRPs and OTI employ Translational Officers or scouts who meet with faculty about their programs (blue), help manage their TRP-funded projects, and report back to OTI and the TRPs on the progress. Overall, the TRPs receive over 100 formal letters of interest over a typical year and they fund up to 30 projects a year at a cost of $2.5 million in direct expenses to projects. While many universities have bundles of innovation branded funding programs, a key feature of our collective approach is that all TRPs are geared first toward protecting IP and product development, and second towards publishing or getting additional grant support. Other agreed upon features are professional project management for all funded projects, specific milestones for each project included in the award, and sharing of resources, ideas, and information through the OTI meetings. This includes publicizing upcoming RFA announcements, sharing reviewer cohorts and review results across programs, and co-funding projects.

OTI meeting attendees hail from the Technology Transfer Office, innovation programs across campus, and the affiliate hospitals. Agenda items include general updates on funding programs such as Coulter and CAHH, training and education programs such as C3i (the Coulter entrepreneurship training program attached to its funding) and i-Corps, special announcements, awards, licenses, and news. OTI’s success relies on these meetings to share resources, create opportunities and allow free flow of information. Without the OTI meetings, we would miss critical opportunities to develop our TRPs.

### Mentoring and Entrepreneurship

As OTI refined its alignment and coordination approach to translation and innovation, an increased need for mentoring individuals who could help in the process became evident. In 2014, with support from an interested Alumnus, we completed the MIT Venture Mentoring Outreach Program and established CWRU Venture Mentor Program (CVMP). We created CVMP to provide CWRU entrepreneurs with unbiased, confidential business advice in a safe and conflict-free environment, in order to help develop, inspire, and empower their pursuit of commercial opportunities in the biomedical space. It is part of our mission to deliver more opportunities to exit technologies from labs into patients and increase the value of the researcher and the venture. CVMP provides team mentoring to students, post-docs, and research associates with translational technologies in the biomedical and healthcare space. We source our mentors from a pool of local and national experts with a wide range of careers and industry expertise. The program is housed within CTSC to provide a broad base for expansion and inclusion.

Further refinements of the approach, which also provides a feeder program for CVMP, led to the formation of the Translational Fellows Program (TFP). The TFP seeks to train individuals in entrepreneurship and the translation of innovation into commercial ventures by connecting them to programs and workshops around campus while protecting time for entrepreneurial activities. Eligible graduate students, post-docs, and research associates receive 20% protected time for a year to pursue entrepreneurial activities within their lab or company. These activities include monthly meetings with themed discussion topics, regional seminars and workshops to learn innovation and entrepreneurial skills, and time devoted to developing their translational research project (i.e. customer discovery, value proposition refinement, creating business plans, etc). Each fellow also undergoes an i-Corps@NCATS program, which is a 5-week shortened version of the iCorps national programs. The pilot year was hugely successful and resulted in four new companies formed, among other successes. The engagement of both trainees and their PIs with the program was very high.

### Benchmarking and Challenges

In relation to other models of academic entrepreneurship and innovation, and with limited capital to develop programing, our office used the national Coulter model to build the foundation for the innovation ecosystem. The staff support system, external review boards, close program management, and funding support were key aspects of building the Council to Advance Human Health (CAHH). The NIH Center for Accelerated Innovation (NCAI) also used parts of the Coulter model for the 7 years of the pilot grant. It was highly successful in the specific vertical of cardiovascular biomedicine (HLBI) [10]. In fact, as one of only three NCAI programs across the country, the best practices were viewed extremely favorably by NIH program reviewers [10]. As a partner institution, we provided project managers and staff support for Case applicants. We also had robust participation in the i-Corps@Ohio training provided by NCAI. Most significantly, we coordinated the matching funds needed to access NCAI funds by utilizing the CAHH, Coulter, and other translational research program funds, through OTI meetings to determine which programs would be the most successful. This had an additional benefit of standardizing all of the translational research programs within the ecosystem, which led to better communication and faster exits. With the combination of national benchmarking through Coulter and NCAI, and the entrepreneurial training through i-Corps@Ohio and i-Corps@NCATS, the Council to Advance Human Health has seen incredible success (Table 2). The rate of CAHH licensing over 3 years is 45–50%, surpassing the national Coulter 3 year licensing rate of 33%.

After exiting from the university, start-ups face different challenges. The regional ecosystem is attempting to address these, especially the issue of limited incubator space. The Cleveland Innovation Project is creating new incubator space to accommodate the rising demand, and local groups such as JumpStart, Inc. and Bounce are providing business and management support. There is a strong need for bolder investment from the investment community, which has been risk averse in the region. Seed funding for start-ups is becoming more available with the support of the Ohio Third Frontier and regional seed funds currently developing.

## Conclusion

Overall, our ecosystem is thriving due in our view to: 1) disciplined investment in industry relevant technologies, 2) continuous scouting of investigator laboratories, 3) focus on building up local entrepreneurs in our academic community, and 4) emphasis on a culture change that supports a cycle of discovery, translation, and commercialization. Our framework illustrated in Fig. 2 keeps us on the path and allows new connections to be made as we mature further. We believe these key attributes provide a framework whereby research institutions looking to become innovation hubs can create a new culture of academic entrepreneurship.
